# Identification and Validation of DEPDC1B as an Independent Early Diagnostic and Prognostic Biomarker in Liver Hepatocellular Carcinoma

**DOI:** 10.3389/fgene.2021.681809

**Published:** 2022-01-13

**Authors:** Xiaoyan Fan, Junye Wen, Lei Bao, Fei Gao, You Li, Dongwei He

**Affiliations:** ^1^ Department of Oncology, Hebei General Hospital, Shijiazhuang, China; ^2^ Department of Hepatobiliary Surgery, Hebei General Hospital, Shijiazhuang, China; ^3^ Laboratory of Pathology, Hebei Cancer Institute, The Fourth Hospital of Hebei Medical University, Shijiazhuang, China

**Keywords:** DEPDC1B, prognosis, diagnosis, biomarker, ROC curve, liver hepatocellular carcinoma

## Abstract

Liver hepatocellular carcinoma (LIHC) is one of the most lethal tumors worldwide, and while its detailed mechanism of occurrence remains unclear, an early diagnosis of LIHC could significantly improve the 5-years survival of LIHC patients. It is therefore imperative to explore novel molecular markers for the early diagnosis and to develop efficient therapies for LIHC patients. Currently, DEPDC1B has been reported to participate in the regulation of cell mitosis, transcription, and tumorigenesis. To explore the valuable diagnostic and prognostic markers for LIHC and further elucidate the mechanisms underlying DEPDC1B-related LIHC, numerous databases, such as Oncomine, Gene Expression Profiling Interactive Analysis (GEPIA), UALCAN, Kaplan-Meier plotter, and The Cancer Genome Atlas (TCGA) were employed to determine the association between the expression of DEPDC1B and prognosis in LIHC patients. Generally, the DEPDC1B mRNA level was highly expressed in LIHC tissues, compared with that in normal tissues (*p* < 0.01). High DEPDC1B expression was associated with poor overall survival (OS) in LIHC patients, especially in stage II, IV, and grade I, II, III patients (all *p* < 0.05). The univariate and multivariate Cox regression analysis showed that DEPDC1B was an independent risk factor for OS among LIHC patients (HR = 1.3, 95% CI: 1.08–1.6, *p* = 0.007). In addition, the protein expression of DEPDC1B was validated using Human Protein Atlas database. Furthermore, the expression of DEPDC1B was confirmed by quantitative real-time polymerase chain reaction (qRT-PCR) assay using five pairs of matched LIHC tissues and their adjacent noncancerous tissues. The KEGG pathway analysis indicated that high expression of DEPDC1B may be associated with several signaling pathways, such as MAPK signaling, the regulation of actin cytoskeleton, p53 signaling, and the Wnt signaling pathways. Furthermore, high DEPDC1B expression may be significantly associated with various cancers. Conclusively, DEPDC1B may be an independent risk factor for OS among LIHC cancer patients and may be used as an early diagnostic marker in patients with LIHC.

## Introduction

Liver hepatocellular carcinoma (LIHC) is the most common type of lethal cancer and is the fourth leading cause of death among cancer patients worldwide (Bray et al., 2018; Villanueva, 2019). The World Health Organization estimates that more than 1 million patients will die from LIHC in 2030 (Villanueva, 2019). Several risk agents, such as chronic infection with hepatitis B virus (HBV) or hepatitis C virus (HCV), and exposure to alcohol and aflatoxins are significantly involved in the intrinsic mechanisms (Wang et al., 2002; El-Serag and Rudolph 2007). Furthermore, recent studies have established that accumulated genetic alterations, such as somatic mutations, and chromosomal aberrations may be involved in this process (Villanueva, 2019). Somatic mutations in the TERT promoter, which is a recurrent insertion site for the genome of HBV, are the most frequent genetic alterations (approximately 60% of cases) (Schulze et al., 2016). Other mutated genes could affect the cell cycle (TP53, approximately 30% of cases) and WNT signaling (CTNNB1 and AXIN1 occurring in approximately 30 and 10% of cases, respectively) (Villanueva, 2019). In addition, chromatin remodeling (ARID1A and ARID2) may also account for approximately 10 and 5% of LIHC patients, respectively (Villanueva, 2019). Though the molecular mechanisms of LIHC remain far from being fully understood, the survival rate of LIHC patients could be improved by more than 50% with early detection of hepatocellular carcinoma (Kim et al., 2016; Lu et al., 2020). Conversely, the early diagnosis of LIHC is far from satisfactory, hence the exploration of novel molecular markers for early diagnosis and therapies is of great value for LIHC patients.

DEP domain-containing protein 1B (DEPDC1B), which is located at chromosome 5 (5q12.1), was initially discovered by mRNA expression profiling in MDA-MB231 human breast cancer cells (Boudreau et al., 2007). DEPDC1B contains two conserved domains: the DEP domain and the RhoGAP domain. The DEP domain is a globular domain containing about 90 amino acids, and was named from three proteins: *Drosophila* disheveled, *Caenorhabditis elegans* EGL-10, and *mammalian* Pleckstrin (Wong et al., 2000; Wharton, 2003). Being more than just a membrane anchor, the DEP domain could negatively interact with charged phospholipids located in membranes to activate Wnt signaling (Sokol, 2000). In addition, the DEP domain could interact directly with the G protein-coupled receptors to regulate GPCR signaling pathways (Ballon et al., 2006; Chen and Hamm, 2006). Moreover, the RhoGAP domain is a key participator in Rho GTPase signaling (Martemyanov et al., 2003). As a protein accumulating during G2 phase, Marchesi et al. have reported that the role of DEPDC1B in coordinating de-adhesion and cell-cycle progression at mitotic entry (Marchesi et al., 2014). Furthermore, increasing evidence in recent years suggests that the overexpression of DEPDC1B is associated with tumor aggressiveness and poor prognosis in cancers, such as oral cancer (Su et al., 2014), malignant melanoma (Xu et al., 2019), glioblastoma (Chen et al., 2020), non-small cell lung (Yang et al., 2014), and pancreatic cancers (Mishra et al., 2019; Liu et al., 2020). These findings strongly suggest that DEPDC1B could potentially contribute to tumorigenesis. However, the role of DEPDC1B in LIHC remains unclear.

In the present study, public databases were used to analyze the correlation between the expression of DEPDC1B and patient diagnostic and prognosis for LIHC. In addition, the results were confirmed by quantitative real-time polymerase chain reaction (qPCR) assay, and the findings from the study suggest that DEPDC1B may have a utility as a potential biomarker for the diagnosis and prognosis in LIHC patients.

## Materials and Methods

### Oncomine Database Analysis

The expression level of DEPDC1B in LIHC was determined by Oncomine database analysis (https://www.oncomine.org/resource/login.html) (Rhodes et al., 2007). The thresholds (*p* ≤ 0.0001, fold change: 2, and gene rank: Top 10%) were considered statistically significant.

### UALCAN Database Analysis

UALCAN is a comprehensive, use-friendly, and interactive web resource for analyzing cancer OMICS data (http://ualcan.path.uab.edu/index.html). UALCAN is designed to provide easy access to publicly available cancer OMICS data (TCGA, MET500, and CPTAC databases), allowing users to identify biomarkers of interest (Chandrashekar et al., 2017). In this study, DEPDC1B expression was analyzed from the TCGA database and *p* < 0.01 was considered statistically significant.

### TCGA Database Analysis

Gene expression data and patient data for LIHC were downloaded from the Genomic Data Commons (GDC) data portal (https://portal.gdc.cancer.gov/) using the GDC data transfer tool. Gene expression data were analyzed using R (version: 3.6.1) with related R packages. Clinical parameters, such as age, gender, survival, and tumor grade and stage were extracted from the patient data and then matched to each patient using a PERL script.

### GEPIA Database Analysis

The Gene Expression Profiling Interactive Analysis (GEPIA) platform (http://gepia.cancer-pku.cn/) is a newly developed interactive web server for analyzing RNA sequencing expression data for 9,736 tumors and 8,587 normal samples from TCGA and the Genotype-Tissue Expression database projects, using a standard processing pipeline (Tang et al., 2017). The database was used to evaluate DEPDC1B expression in LIHC patients. In the survival analysis, the threshold was determined according to the following values: group cutoff: median; cutoff-high (%): 50; cutoff-low (%): 50.

### Kaplan-Meier Plotter Database Analysis

Based on the Kaplan Meier plotter (http://kmplot.com/analysis/) (Nagy et al., 2018), the correlation between DEPDC1B mRNA expression and survival in LIHC was analyzed using RNA-seq data. The patients were divided into low and high expression groups according to median expression, and the cutoff value was set to “auto select”.

### ROC Curve Generation

Receiver operating characteristic (ROC) curves were generated to evaluate the diagnostic value of DEPDC1B using IBM SPSS Statistics 26. And the area under the curve (AUC) was also determined and showed in the panel.

### KEGG Analysis

To identify the potential mechanisms of DEPDC1B expression in LIHC, KEGG analysis was performed to detect whether a priori defined set of genes showed statistically significant differential expression between the high and low DEPDC1B expression groups using GSEA (Subramanian et al., 2005). Gene sets with a normal *p*-value < 0.05 and false discovery rate (FDR) < 0.05 were significantly enriched.

### Immunohistochemistry Validation of DEPDC1B Expression Using Human Protein Atlas Database

To further confirm the expression level of DEPDC1B in LIHC tissues, DEPDC1B protein expression was analyzed in clinical specimens using The Human Protein Atlas (https://www.proteinatlas.org/).

### RNA Extraction and qPCR Assay

qPCR was performed to determine the expression of DEPDC1B mRNA in LIHC and their adjacent tissues. Briefly, total RNA from the surgically obtained paired tissues (*n* = 5) was isolated using TRI Reagent RNA Isolation Reagent (Sigma-Aldrich) according to the manufacturer’s instructions. A reverse transcription system was used to obtain the first-strand template Complementary DNA (cDNA). The primer sequences were used as follows: DEPDC1B: 5′- GAG​CTA​CCA​GGC​TGT​GGA​AT-3′ (forward) and 5′- GCC​GAA​GTT​TTG​ACT​GCA​CC -3′ (reverse); GAPDH: 5′-CCATGTTCGTCATGGGTGTGAACCA-3′(forward) and 5′-GCCAGTAGAGGCAGGGATGATGTTC-3′(reverse) (Li et al., 2020; Zhang et al., 2020). The expression of GAPDH was considered as an internal control. Each reaction was performed in triplicate. The study was approved by the Institute Research Ethics Committee at the Fourth Hospital of Hebei Medical University.

### Statistical Analysis

Two-tailed *p* values less than 0.05 were considered statistically significant. TCGA-associated expression and prognosis analyses were conducted using R software (version 3.6.1). The univariate Cox analysis was used to select potential prognostic factors, and multivariate Cox analysis was performed to verify the correlations between DEPDC1B expression and survival, along with other clinical features.

## Results

### High DEPDC1B mRNA Expression in LIHC

**FIGURE 1:**
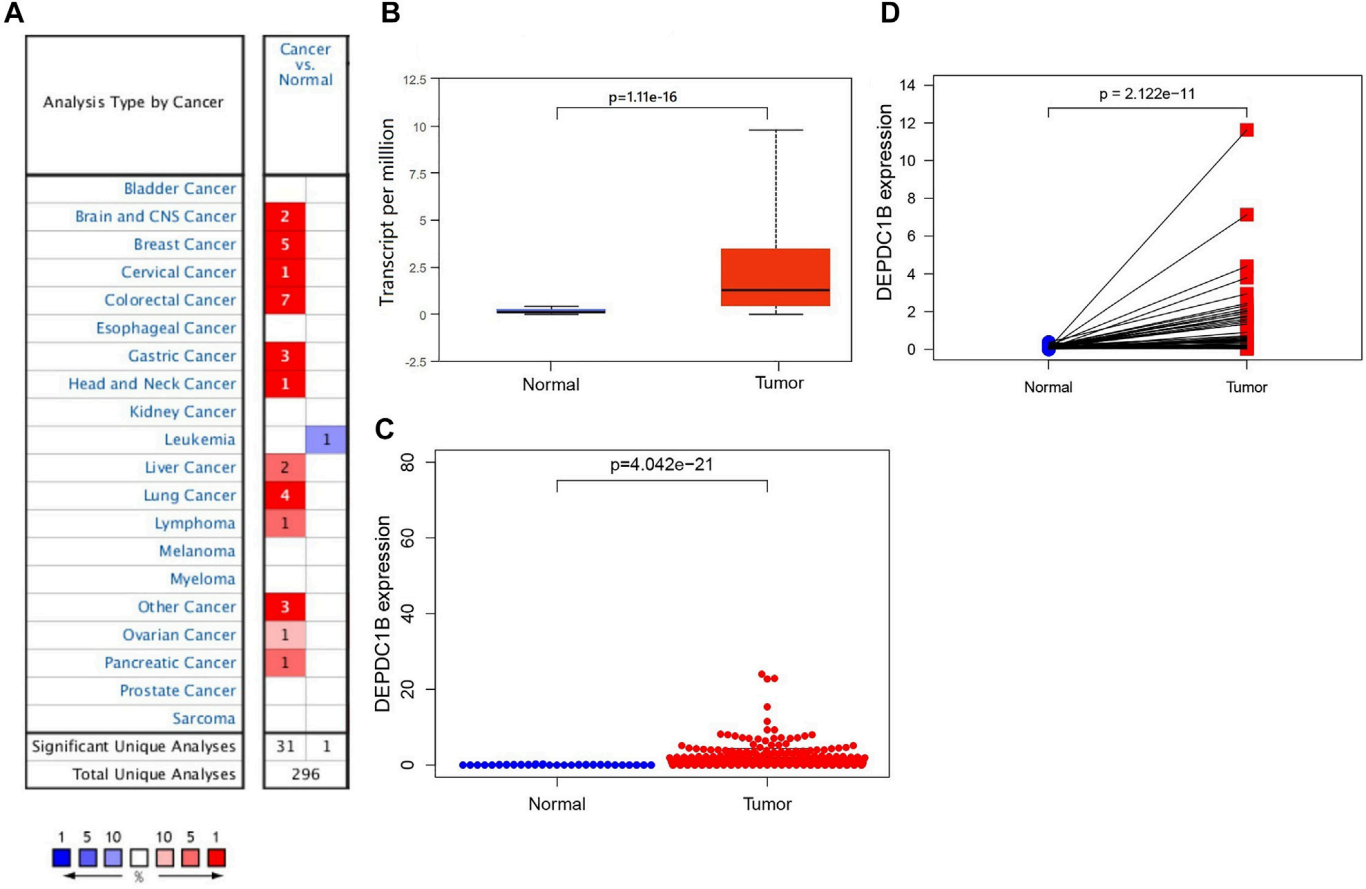
DEPDC1B expression in LIHC tissues using online databases. **(A)** Expression of DEPDC1B (normal compared with cancer tissue) were analyzed using Oncomine database. The table shows the number of significant analyses across the whole Oncomine database (715 datasets and 86.733 samples). DEPDC1B shows high/low expression ratio of 31/1 from 32 analyses. The cell color is determined by the best gene rank percentile for the analyses within the cell. The number in each cell represents the number of analyses that meet the threshold within those analysis and cancer types. The gene rank was analyzed by percentile of target gene in the top of all genes measured in each research. **(B)** UALCAN database. The number of patients in normal and primary tumor groups was 50 and 371, respectively. *p* = 1.11e-16. **(C)** TCGA database using R script. Normal (*n* = 50), Tumor (*n* = 374). *p* = 4.042e-21. **(D)** DEPDC1B expression in a paired comparison of LIHC and their adjacent tissues. Data were extracted from the TCGA database. *n* = 50. *p* = 2.122e-11.

Oncomine and UALCAN online databases were used to determine the expression levels of DEPDC1B mRNA in LIHC and their normal tissues. Both databases showed higher levels of DEPDC1B expression in LIHC tissues when compared with normal tissues (all *p* < 0.01; Figures 1A,B). For validation, we downloaded RNA-seq data for LIHC from TCGA database and analyzed the expression of DEPDC1B using R. Figure 1C indicates that when compared with that in normal tissues, the DEPDC1B expression was significantly upregulated in TCGA LIHC tissues (*p* = 4.042e-21; Figure 1C). Additionally, the pair-wise comparison of TCGA LIHC tissues and their adjacently matched tissues revealed a significantly higher level of DEPDC1B expression in the former (*p* = 2.122e-11; Figure 1D). These results suggest that the expression of DEPDC1B is highly elevated in LIHC when compared with normal tissues.

### Prognostic Potential of DEPDC1B in LIHC

**FIGURE 2:**
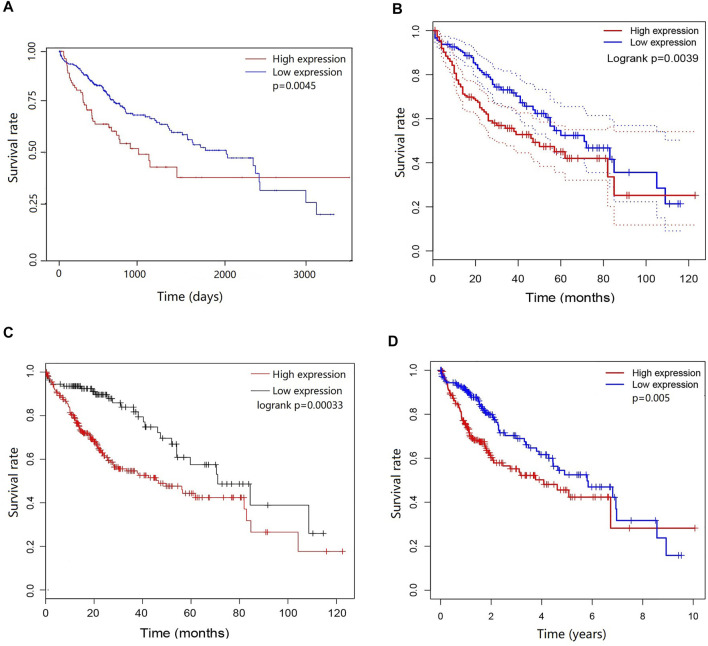
Prognostic potential of DEPDC1B in LIHC. Four online tools were used to evaluate the relationships between DEPDC1B expression and patient survival. **(A)** OS in UALCAN database, the samples were divided into high DEPDC1B expression (*n* = 88) and low/medium DEPDC1B expression (*n* = 277) groups according to the median of DEPDC1B expression. *p* = 0.0045 **(B)** OS in GEPIA database. The samples were divided into high DEPDC1B expression (*n* = 181) and low DEPDC1B expression (*n* = 181) groups according to the median of DEPDC1B expression. *p* = 0.0039 **(C)** OS in Kaplan-Meier Plotter database. The number of patients in high and low groups was 252, 112, respectively. *p* = 0.00033 **(D)** OS in TCGA database, *p* = 0.005. The samples were divided into high DEPDC1B expression (*n* = 260) and low DEPDC1B expression (*n* = 110) groups according to the median of DEPDC1B expression. *p* = 0.005. OS: overall survival.

To determine the potential prognostic significance of the expression of DEPDC1B in LIHC, UALCAN, GEPIA, Kaplan-Meier Plotter, and R script were used to evaluate the relationships between DEPDC1B expression and the survival rate of patients. Figure 2 shows that high DEPDC1B mRNA expression was significantly associated with poor overall survival (OS) in LIHC patients using UALCAN (*p* = 0.0045; Figure 2A), GEPIA (logrank *p* = 0.0039, HR = 1.7; Figure 2B), Kaplan-Meier Plotter [logrank *p* = 0.00033, HR = 2.14 (1.4–3.28); Figure 2C] analyses. In addition, clinical data for LIHC cases were downloaded from TCGA database, and the OS was subsequently analyzed using R (Table 1; Figure 2D). Consistent with previous findings reported above, high DEPDC1B expression was significantly and negatively associated with the survival of patients with TCGA LIHC (*p* = 0.005; Figure 2D). These results suggest that high DEPDC1B expression could lead to a poor prognosis in patients with LIHC.

**TABLE 1:** Clinical characteristics of the 349 LIHC patients downloaded from the TCGA database.

Characteristic	N (%)
Age (years)	
≤60	174 (49.86)
>60	175 (50.14)
Gender	
Female	110 (31.52)
Male	239 (68.48)
Stage	
I	173 (49.57)
II	85 (24.36)
III	86 (24.64)
IV	5 (1.43)
T classification	
T1	175 (50.14)
T2	87 (24.93)
T3	77 (22.06)
T4	10 (2.87)
Survival status	
Death	113 (32.39)
Survival	236 (67.61)

### Association Between DEPDC1B Expression and Clinical Characteristics of LIHC Patients

Since the expression of DEPDC1B was negatively associated with the prognosis of LIHC patients, to better understand the relevance, and mechanisms of DEPDC1B in LIHC, we determined the relationship between the expression of DEPDC1B and the clinicopathological characteristics of LIHC patients using the Kaplan-Meier plotter tool. DEPDC1B expression was negatively associated with OS in female and male patients (*p* = 0.019, *p* = 4.42e-04, respectively; Table 2; Supplementary Figure S1), and patients from white and Asian races (*p* = 0.014, *p* = 8.9e-08, respectively). High DEPDC1B mRNA expression has a significant correlation with low OS in stage II, IV, and grade I, II, III patients (*p* = 0.018, *p* = 0.0067, *p* = 0.0021, *p* = 0.0043, *p* = 0.022, respectively). Also, similar significant results were found between the expression of DEPDC1B and Relapse Free Survival (RFS) in gender, race stage, and grade (Table 2; Supplementary Figure S1; all *p* < 0.05). Interestingly, in patients with risk factors (especially alcohol consumption and hepatitis virus), the expression of DEPDC1B had no significant impact on the OS of patients with LIHC (*p* = 0.059, *p* = 0.31, respectively). These results suggest that high DEPDC1B expression significantly affects the OS and RFS of LIHC patients exhibiting most clinical characteristics.

**TABLE 2:** Correlation of DEPDC1B expression and clinical prognosis in LIHC with different clinical characteristics by Kaplan-Meier plotter.

Clinical characteristics	OS		RFS	
	HR (95%CI)	Logrank P	HR (95%CI)	Logrank P
Gender				
Female	1.96 (1.11–3.46)	0.019*	2.82 (1.54–5.16)	0.00046***
Male	2.2 (1.4–3.44)	4.42e-04***	1.69 (1.08–2.65)	0.02*
Race				
White	1.86 (1.13–3.08)	0.014*	2.13 (1.27–3.58)	0.0035**
Asian	4.52 (2.47–8.27)	8.9e-8***	2.51 (1.3–4.83)	0.0044**
Stage				
1	1.78 (0.97–3.27)	0.06	1.63 (0.94–2.81)	0.078
2	3.08 (1.15–8.23)	0.018*	−1.9 (0.79–4.58)	−0.14
3	2.23 (1.23–4.05)	0.0067**	2.23 (1.23–4.05)	0.0067**
Grade				
1	5.02 (1.66–15.13)	−0.0021**	−3.08 (0.7–13.66)	−0.12
2	2.53 (1.31–4.88)	0.0043**	−2.13 (1.21–3.74)	−0.0075**
3	1.98 (1.09–3.61)	0.022*	1.78 (1.02–3.1)	0.04*
Vascular invasion				
None	1.87 (1.12–3.14)	0.016*	1.44 (0.88–2.361)	0.15
Micro	1.88 (0.79–4.46)	0.15	1.88 (0.79–4.46)	0.15
Risk factors				
Alcohol consumption				
Yes	1.84 (0.97–3.5)	0.059	2.6 (1.41–4.79)	0.0015**
None	2 (1.26–3.18)	0.0027**	1.63 (1.05–2.55)	0.029*
Hepatitis virus				
Yes	1.39 (0.73–2.67)	0.31	1.27 (0.74–2.15)	0.39
None	3.68 (1.97–6.87)	1.4e-05***	3.92 (2.01–7.62)	1.7e-05***

- values indicate the sample number too low for meaningful analysis.

*values indicate *p* < 0.05, ** values indicate *p* < 0.01, *** values indicate *p* < 0.001.

### High DEPDC1B Expression is an Independent Risk Factor for OS Among LIHC Patients

**FIGURE 3:**
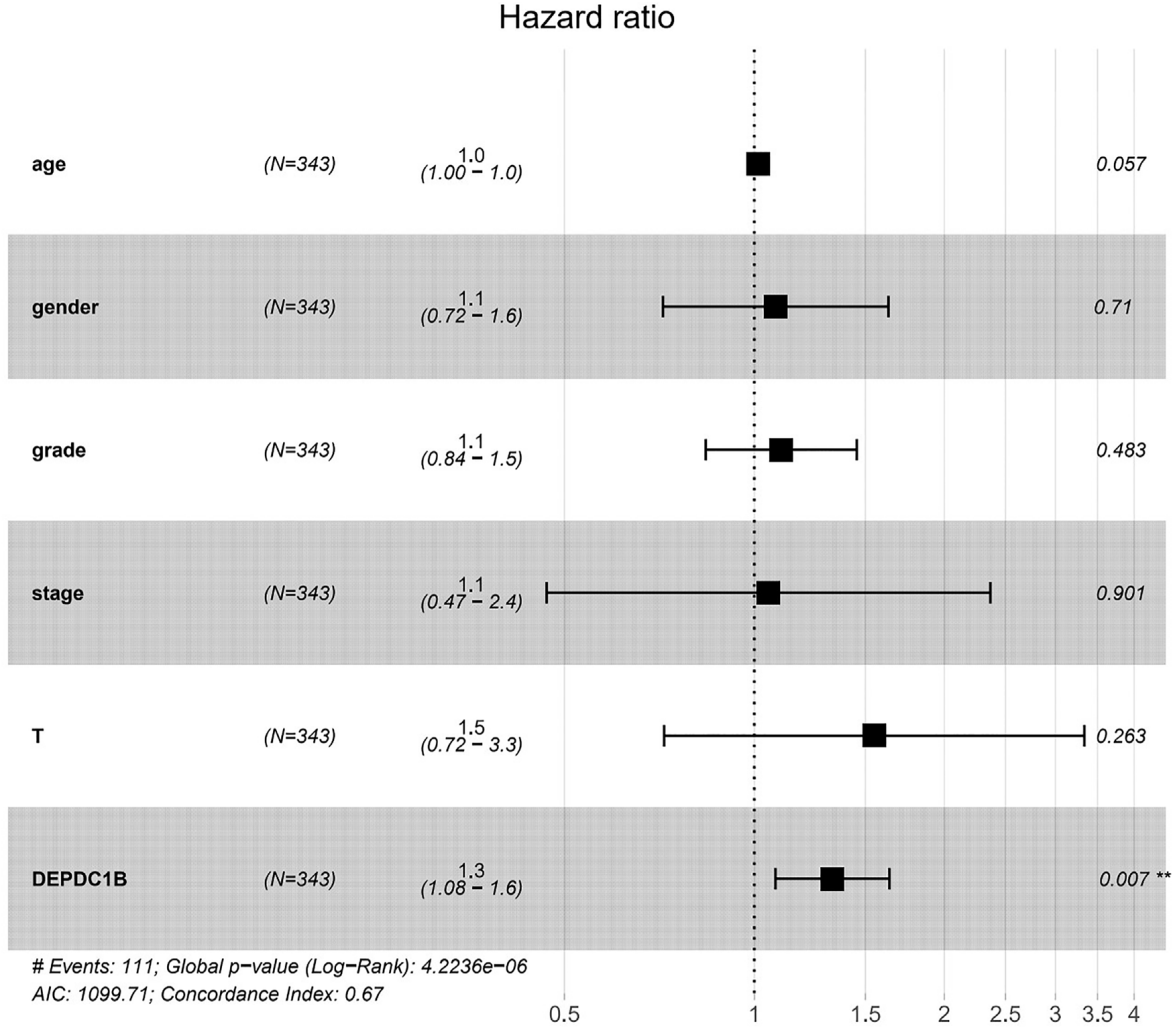
DEPDC1B is an independent risk factor for OS among LIHC patients using multivariate Cox analysis. **p = 0.007.

To investigate whether DEPDC1B is an independent risk factor for OS in LIHC patients, univariate and multivariate Cox analyses were performed using an R script. In the univariate Cox analysis, tumor stage, T classification, and DEPDC1B expression were all independent risk factors for OS (*p* = 1.12e-06, 5.82e-07, and 0.01, respectively; Table 3). In the multivariate Cox analysis, only the expression of DEPDC1B was found to be an independent risk factor for OS (*p* = 0.007, HR = 1.33, 95% CI: 1.08–1.64; Table 3; Figure 3). These findings indicate that the expression of DEPDC1B expression could be an independent risk factor for the OS of LIHC patients.

**TABLE 3:** Univariate and multivariate analysis of the correlation of DEPDC1B expression with OS among LIHC patients.

Parameter	Univariate analysis			Multivariate analysis		
	HR	95% CI	*p*-value	HR	95% CI	*p*-value
Age	1.01	0.995–1.025	0.177	1.015	1.000–1.031	0.057
Gender	0.82	0.557–1.209	0.317	1.081	0.716–1.632	0.710
Grade	1.12	0.868–1.446	0.382	1.104	0.838–1.453	0.483
Stage	1.67	1.400–2.056	1.12e-06***	1.053	0.468–2.367	0.901
T classification	1.65	1.357–2.011	5.82e-07***	1.549	0.719–3.336	0.263
DEPDC1B	1.07	1.014–1.124	0.01*	1.330	1.080–1.637	0.007**

*values indicate *p* < 0.05, ** values indicate *p* < 0.01, *** values indicate *p* < 0.001.

### Diagnostic Value of DEPDC1B Expression in LIHC Patients

**FIGURE 4:**
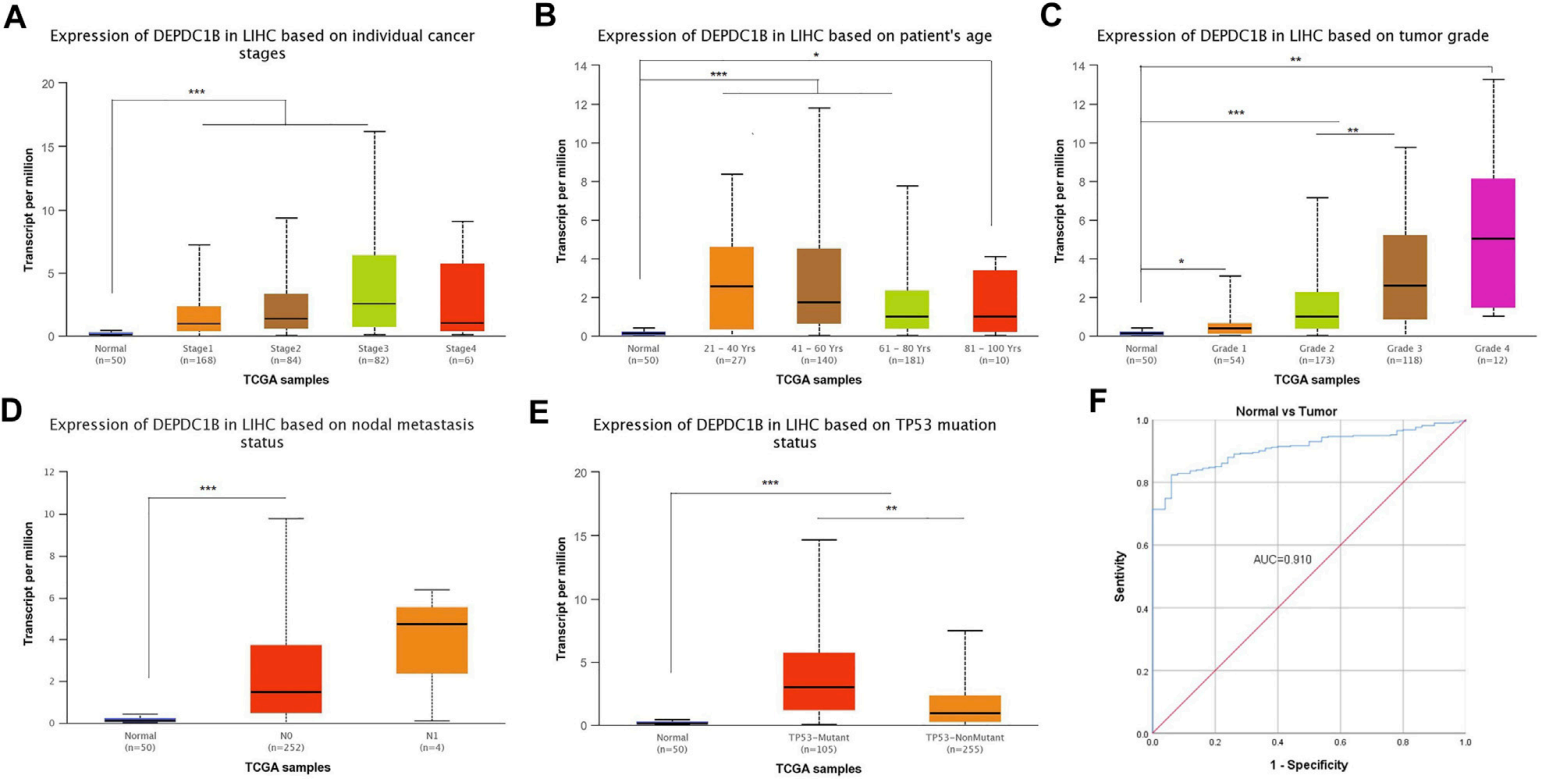
Diagnostical potential of DEPDC1B expression in LIHC. Association between DEPDC1B expression and clinical characters: **(A)** cancer stage. Normal vs Stage 1, *p* = 1.62e-12; Normal *vs.* Stage 2, *p* = 3.44e-06; Normal *vs.* Stage 3, *p* = 2.59e-07; Stage 1 *vs.* Stage 3, *p* = 2.15e-03. **(B)** patient’s age. Normal *vs.* Age (21–40 Years), *p* = 9.70e-05; Normal *vs.* Age (41–60 Years), *p* = 6.92e-11; Normal *vs.* Age (61–80 Years), *p* = 6.81e-10, Normal *vs.* Age (81–100 Years), *p* = r0.046. **(C)** tumor grade. Normal vs Grade 1, *p* = 4.80e-02; Normal vs Grade 2, *p* = 2.85e-12; Normal vs Grade 3, *p* = 8.09e-12; Normal *vs.* Grade 4, *p* = 1.30e-03. Grade 2 *vs.* Grade 3, *p* = 3.30e-03. **(D)** LIHC nodal metastasis status. Normal *vs.* N0, p < 1E-12. **(E)** TP53 mutation status. Normal *vs.* TP53-Mutant, p < 1E-12; Normal *vs.* TP53-NonMutant, *p* = 1.99e-11; TP53-Mutant *vs.* TP53-NonMutant, *p* = 1.94e-03. **(F)** Diagnosis value of DEPDC1B using ROC analysis. AUC: area under ROC curve. Student’s t test was used to generate a *p*-value. *p < 0.05, **p < 0.01, ***p < 0.001.

Figures 4A–E indicates the observance of significant differences in the expression of DEPDC1B and the clinical characteristics (Normal *vs.* Stages, Normal *vs.* age, Normal *vs.* grade, Normal *vs.* nodal metastasis, and Normal *vs.* TP53 mutation status, all *p* < 0.001) in LIHC patients. The results above indicated that the expression of DEPDC1B may be a potential diagnostic biomarker for LIHC. To further elucidate the diagnostic value of DEPDC1B in LIHC patients, ROC curves were generated using SPSS 26.0. The AUC was 0.91, which strongly suggested that the level of DEPDC1B mRNA expression might be a strong diagnostic biomarker in LIHC (Figure 4F).

### KEGG Pathway Analysis

**FIGURE 5:**
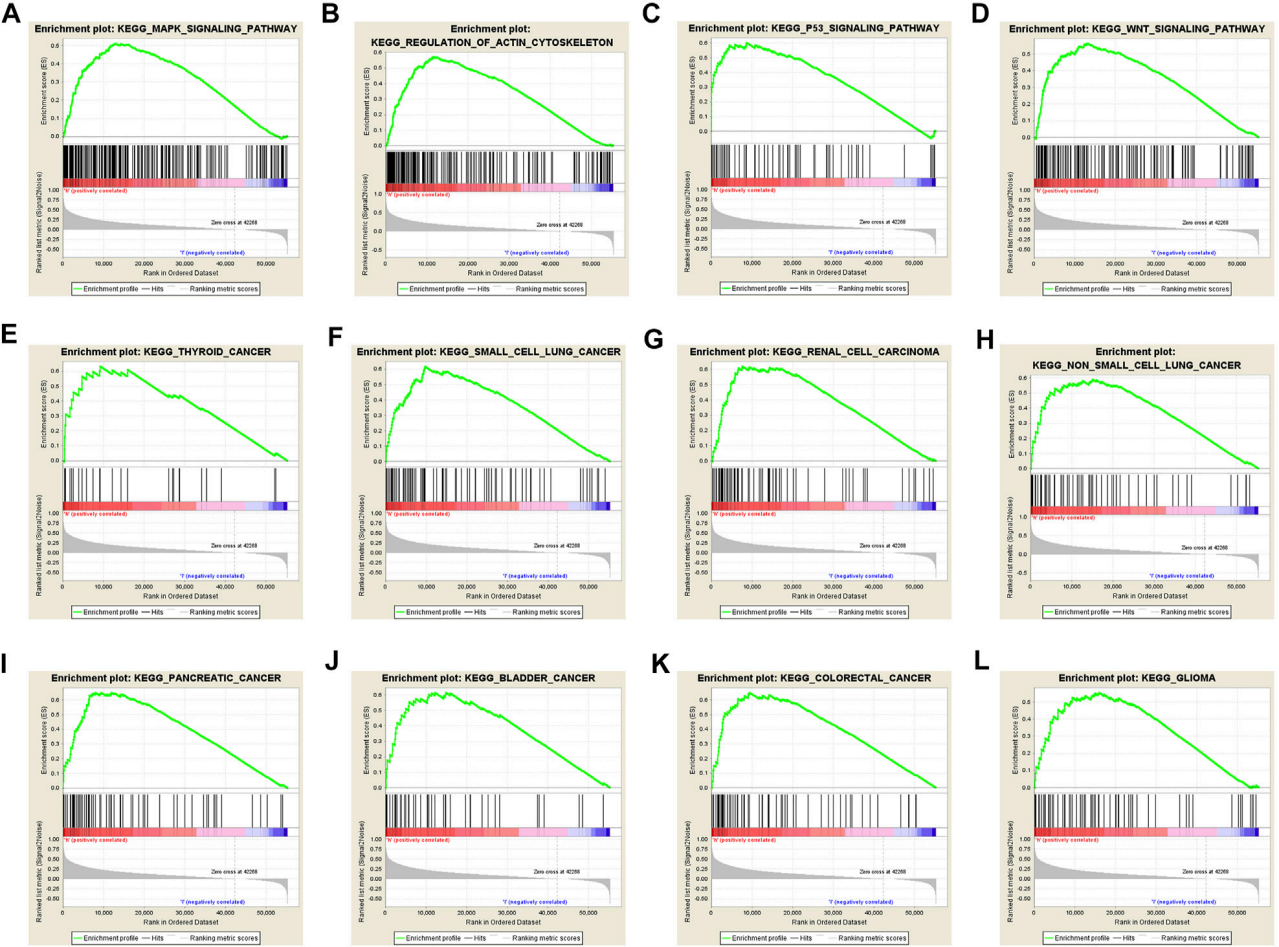
Gene sets enriched in the high DEPDC1B expression phenotype using GSEA 3.0. Panel **(A–D)** showing some verified signaling pathways: **(A)** MAPK signaling pathway **(B)** regulation of actin cytoskeleton **(C)** p53 signaling pathway **(D)** Wnt signaling pathway; Panel **(E–L)** showing some cancer-associated pathways **(E)** Thyroid cancer **(F)** small cell lung cancer **(G)** renal cell carcinoma **(H)** non-small cell lung cancer **(I)** pancreatic cancer **(J)** bladder cancer **(K)** colorectal cancer **(L)** glioma.

To further explore the possible mechanism of DEPDC1B in LIHC, the KEGG analysis was performed to clarify the DEPDC1B-associated signaling pathways. The analysis revealed that 139/178 gene sets are upregulated in the high DEPDC1B expression phenotype, and 39/178 gene sets are upregulated in the low DEPDC1B expression phenotype (Table 4). Gene sets differentially enriched in the high expression of DEPDC1B phenotype included several familiar signaling pathways, such as the MAPK signaling, the regulation of actin cytoskeleton, p53 signaling, and the Wnt signaling pathways (Table 4; Figures 5A–D). Furthermore, the high expression of DEPDC1B may be associated with various cancers (Figures 5E–L).

**TABLE 4:** KEGG pathways in the high DEPDC1B expression phenotype.

Gene set name	NES	NOM p-val	FDR q-val
KEGG_CELL_CYCLE	2.24	0.00	0.00
KEGG_OOCYTE_MEIOSIS	2.20	0.00	0.00
KEGG_BASE_EXCISION_REPAIR	2.11	0.00	0.00
KEGG_PROGESTERONE_MEDIATED_OOCYTE_MATURATION	2.09	0.00	0.00
KEGG_RNA_DEGRADATION	2.08	0.00	0.00
KEGG_UBIQUITIN_MEDIATED_PROTEOLYSIS	2.07	0.00	0.00
KEGG_NUCLEOTIDE_EXCISION_REPAIR	2.02	0.00	0.00
KEGG_P53_SIGNALING_PATHWAY	2.02	0.00	0.00
KEGG_SPLICEOSOME	2.02	0.00	0.00
KEGG_ENDOCYTOSIS	2.00	0.00	0.00
KEGG_PATHWAYS_IN_CANCER	2.00	0.00	0.00
KEGG_DNA_REPLICATION	2.00	0.00	0.00
KEGG_NEUROTROPHIN_SIGNALING_PATHWAY	1.99	0.00	0.00
KEGG_PYRIMIDINE_METABOLISM	1.98	0.00	0.00
KEGG_PURINE_METABOLISM	1.98	0.00	0.00
KEGG_INOSITOL_PHOSPHATE_METABOLISM	1.96	0.00	0.00
KEGG_ADHERENS_JUNCTION	1.96	0.00	0.00
KEGG_COLORECTAL_CANCER	1.95	0.00	0.00
KEGG_BASAL_TRANSCRIPTION_FACTORS	1.94	0.00	0.00
KEGG_GLYCOSYLPHOSPHATIDYLINOSITOL_GPI_ANCHOR_BIOSYNTHESIS	1.93	0.00	0.01
KEGG_PHOSPHATIDYLINOSITOL_SIGNALING_SYSTEM	1.93	0.00	0.00
KEGG_REGULATION_OF_ACTIN_CYTOSKELETON	1.93	0.00	0.00
KEGG_HOMOLOGOUS_RECOMBINATION	1.92	0.00	0.01
KEGG_GNRH_SIGNALING_PATHWAY	1.92	0.00	0.00
KEGG_SMALL_CELL_LUNG_CANCER	1.92	0.00	0.00
KEGG_PANCREATIC_CANCER	1.92	0.00	0.00
KEGG_VASOPRESSIN_REGULATED_WATER_REABSORPTION	1.92	0.00	0.00
KEGG_CHRONIC_MYELOID_LEUKEMIA	1.91	0.00	0.01
KEGG_FC_GAMMA_R_MEDIATED_PHAGOCYTOSIS	1.90	0.00	0.01
KEGG_N_GLYCAN_BIOSYNTHESIS	1.88	0.00	0.01
KEGG_WNT_SIGNALING_PATHWAY	1.88	0.00	0.01
KEGG_RENAL_CELL_CARCINOMA	1.87	0.00	0.01
KEGG_ERBB_SIGNALING_PATHWAY	1.86	0.00	0.01
KEGG_NOTCH_SIGNALING_PATHWAY	1.86	0.00	0.01
KEGG_BLADDER_CANCER	1.85	0.00	0.01
KEGG_MISMATCH_REPAIR	1.85	0.00	0.01
KEGG_INSULIN_SIGNALING_PATHWAY	1.85	0.00	0.01
KEGG_LONG_TERM_POTENTIATION	1.83	0.00	0.01
KEGG_THYROID_CANCER	1.82	0.00	0.01
KEGG_MAPK_SIGNALING_PATHWAY	1.82	0.00	0.01
KEGG_PATHOGENIC_ESCHERICHIA_COLI_INFECTION	1.81	0.00	0.01
KEGG_T_CELL_RECEPTOR_SIGNALING_PATHWAY	1.80	0.00	0.01
KEGG_MELANOGENESIS	1.79	0.00	0.01
KEGG_NON_SMALL_CELL_LUNG_CANCER	1.79	0.00	0.01
KEGG_ACUTE_MYELOID_LEUKEMIA	1.79	0.00	0.01
KEGG_TIGHT_JUNCTION	1.79	0.00	0.01
KEGG_MTOR_SIGNALING_PATHWAY	1.78	0.00	0.01
KEGG_GLIOMA	1.78	0.00	0.01
KEGG_AMINOACYL_TRNA_BIOSYNTHESIS	1.78	0.00	0.01
KEGG_TGF_BETA_SIGNALING_PATHWAY	1.78	0.00	0.01
KEGG_VEGF_SIGNALING_PATHWAY	1.77	0.00	0.01
KEGG_PROSTATE_CANCER	1.76	0.01	0.02
KEGG_RIG_I_LIKE_RECEPTOR_SIGNALING_PATHWAY	1.76	0.00	0.02
KEGG_FC_EPSILON_RI_SIGNALING_PATHWAY	1.76	0.00	0.02
KEGG_EPITHELIAL_CELL_SIGNALING_IN_HELICOBACTER_PYLORI_INFECTION	1.76	0.00	0.02
KEGG_APOPTOSIS	1.73	0.00	0.02
KEGG_LEUKOCYTE_TRANSENDOTHELIAL_MIGRATION	1.73	0.00	0.02
KEGG_REGULATION_OF_AUTOPHAGY	1.73	0.01	0.02
KEGG_VIBRIO_CHOLERAE_INFECTION	1.73	0.01	0.02
KEGG_LONG_TERM_DEPRESSION	1.73	0.00	0.02
KEGG_CYTOSOLIC_DNA_SENSING_PATHWAY	1.72	0.01	0.02
KEGG_RNA_POLYMERASE	1.72	0.02	0.02
KEGG_DORSO_VENTRAL_AXIS_FORMATION	1.72	0.01	0.02
KEGG_ENDOMETRIAL_CANCER	1.72	0.01	0.02
KEGG_SNARE_INTERACTIONS_IN_VESICULAR_TRANSPORT	1.68	0.01	0.03
KEGG_GAP_JUNCTION	1.67	0.01	0.03
KEGG_NATURAL_KILLER_CELL_MEDIATED_CYTOTOXICITY	1.66	0.03	0.03
KEGG_NOD_LIKE_RECEPTOR_SIGNALING_PATHWAY	1.65	0.01	0.04
KEGG_GLYCEROPHOSPHOLIPID_METABOLISM	1.65	0.00	0.04
KEGG_SELENOAMINO_ACID_METABOLISM	1.65	0.01	0.04
KEGG_AXON_GUIDANCE	1.65	0.01	0.03
KEGG_ETHER_LIPID_METABOLISM	1.64	0.01	0.04
KEGG_TOLL_LIKE_RECEPTOR_SIGNALING_PATHWAY	1.64	0.02	0.04
KEGG_HEDGEHOG_SIGNALING_PATHWAY	1.63	0.01	0.04
KEGG_CHEMOKINE_SIGNALING_PATHWAY	1.63	0.02	0.04

### Validation of DEPDC1B Protein Expression Level

**FIGURE 6:**
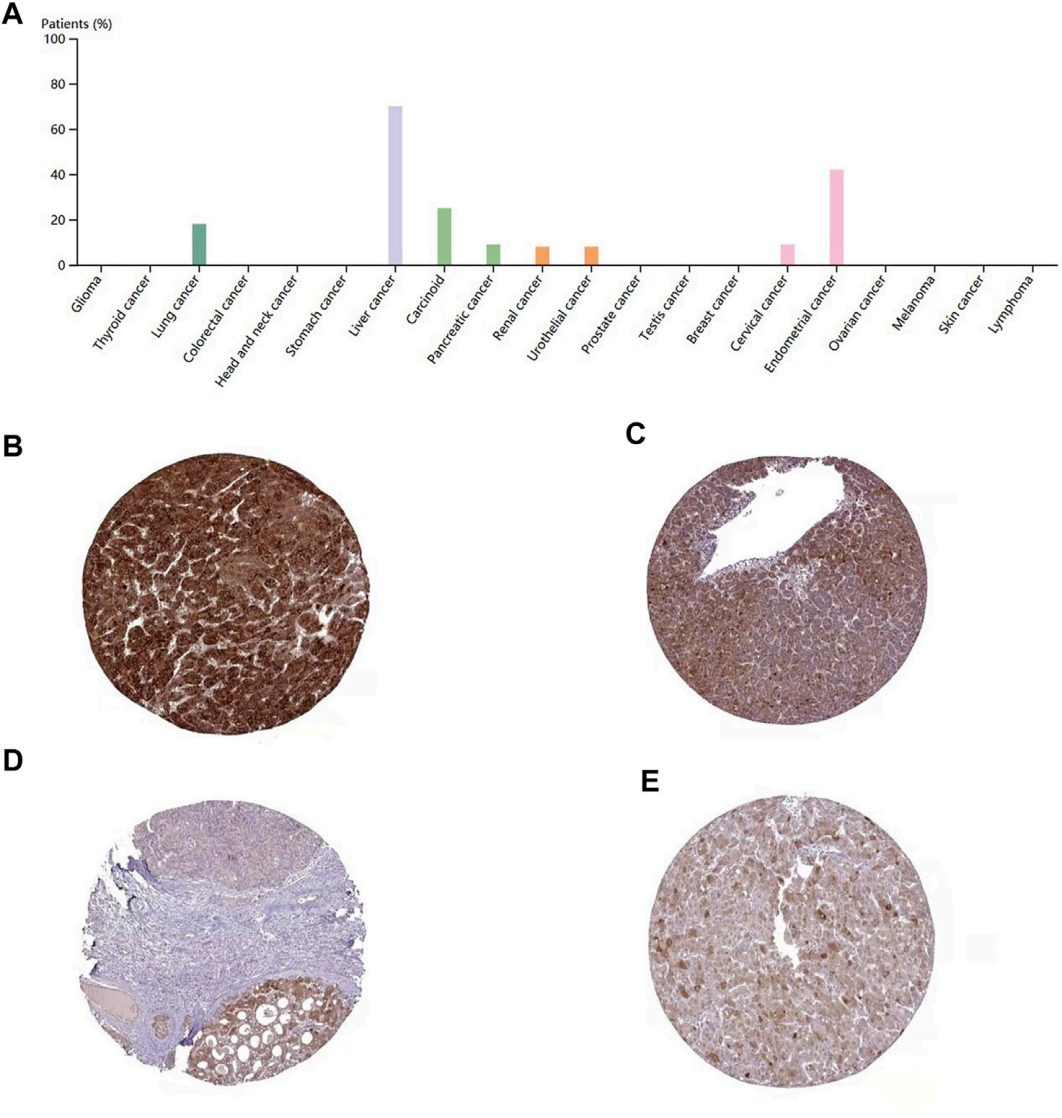
immunohistochemistry of DEPDC1B in various types of cancer **(A)** and LIHC tissues **(B–E)** using HPA072558 antibody. **(A)** Strong cytoplasmic positivity was displayed in several hepatocellular carcinomas and single cases of carcinoma and urothelial cancer. Several endometrial cancers and a few other cancer tissues showed moderate immunoreactivity. The remaining cancer tissues were weakly stained or negative. Tumor cells staining: high expression **(B,C)** and medium expression **(D,E)**; **(B)**Patient id: 3,477, male, age 67; **(C)**: Patient id: 5,032, female, age 58; **(D)** Patient id: 3,196, male, age 65; **(E)** Patient id: 4,823, female, age 25. DEPDC1B was mainly stained in the cytoplasmic/membranous LIHC cells using HPA072558 antibody (Atlas Antibodies Sigma-Aldrich).

To evaluate the protein level of DEPDC1B, immunohistochemistry was analyzed using the Human Protein Atlas database. As indicated in Figure 6A, the DEPDC1B protein was strongly expressed in liver cancer, compared with that in other cancers using HPA072558 antibody (Atlas Antibodies Sigma-Aldrich) (Figure 6A). In addition, the pattern of DEPDC1B expression in LIHC tissues is shown in Figure 6. (strong: Figures 6B,C; medium: Figures 6D,E).

### High Expression of DEPDC1B mRNA in LIHC Tissues

**FIGURE 7:**
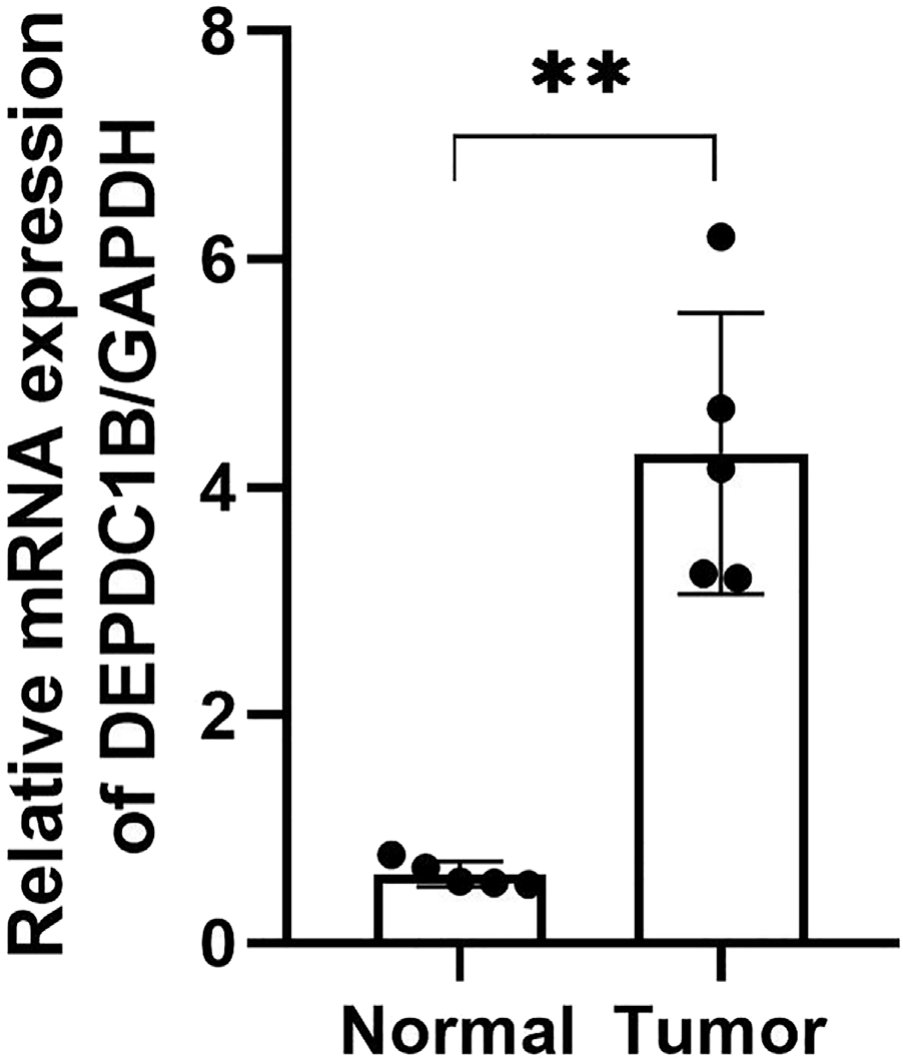
DEPDC1B mRNA expression is frequently upregulated in LIHC tissues. T: LIHC tissues; N: noncancerous tissues. The results were shown with Mean ± SD. N = 5, **p < 0.01.

To confirm the expression of DEPDC1B mRNA in LIHC, we performed qPCR in five pairs of matched LIHC tissues and their adjacent noncancerous tissues, and as shown in Figure 7, the expression of DEPDC1B mRNA was upregulated in LIHC cancer tissues, compared with that in the corresponding noncancerous tissues (*p* < 0.01).

## Discussion

Recently, systemic therapies for LIHC patients are rapidly changing (Villanueva, 2019). Compared with less than 15% of the 5-years survival rate of patients detected at later stages, the early diagnosis of LIHC could result in a survival rate of more than 50%. However, since the value of current diagnostic biomarkers in LIHC is greatly limited (Sun and Zhang, 2020), it becomes paramount to identify novel biomarkers for the treatment of LIHC patients as such biomarkers may help to improve the 5-years survival rate of LIHC patients and further help to establish personalized treatments for each patient. Herein, the current study presented the value of DEPDC1B as a potential biomarker in LIHC.

In 2007, Boudreau et al. reported that DEPDC1B was highly expressed in the placenta and testis with only little expressions in the small intestine and the heart (Boudreau et al., 2007). In the current study, using Human Protein Atlas database, we further confirmed that DEPDC1B was highly expressed in the placenta and testis, with only little expressions in the heart and the small intestine (Supplementary Figure S2). Furthermore, DEPDC1B is highly expressed in the 1) gallbladder and the thymus at the RNA level and 2) gallbladder, lymph node, and tonsil at protein level (Supplementary Figure S3) indicated that DEPDC1B might play an important role in the gallbladder, albeit the detailed mechanism remains unclear.

DEPDC1B plays an important role in the regulation of cell mitosis, transcription, and tumorigenesis (Martemyanov et al., 2003; Marchesi et al., 2014). Increasing evidence suggests that DEPDC1B is associated with various types of human cancers. However, the expression pattern and roles of DEPDC1B in LIHC remain unclear. To evaluate the role of DEPDC1B in LIHC, we used various online tools to observe the expression of DEPDC1B in LIHC. As indicated in Figure 1, the expression of DEPDC1B was higher in LIHC tissues than in normal tissues, and this is consistent with the results in non-small cell lung cancer, oral cancer, malignant melanoma (Xu et al., 2019), bladder cancer (Lai et al., 2020), glioblastoma, and pancreatic cancer (Liu et al., 2020). Using qPCR assay, we confirmed the higher DEPDC1B mRNA expression levels in LIHC than that in the normal tissues (Figure 7). These corresponding results further verified the expression of DEPDC1B mRNA is upregulated in LIHC tissues. Notably, some studies demonstrated that the overexpression of DEPDC1B could be used as a prognostic biomarker to predict the outcomes of patients with prostate and non-small cell lung cancers (Yang et al., 2014; Bai et al., 2017). Consistent with these previous reports, the present study indicated that the expression of DEPDC1B was negatively associated with patient prognosis (Figure 2), suggesting that the expression of DEPDC1B may be an invaluable prognostic biomarker for this disease. In addition, the univariate and multivariate Cox analyses further indicated the expression of DEPDC1B mRNA may be a useful biomarker in the prognosis of LIHC cancer (Figure 3). As aforementioned, this study was mainly focused on early-stage/grade LIHC and comparisons with normal tissues. As indicated in Figure 4, significant differences were observed in the DEPDC1B expression between the normal and tumor stage/grade cells. These exciting results further verified that DEPDC1B may be used as a valuable diagnostic biomarker of early-stage/grade LIHC. Generally, these multifaceted results strongly suggest that DEPDC1B may be an early diagnostic and prognostic biomarker in LIHC.

DEPDC1B either directly or indirectly affects the prognosis of patients with LIHC, however, the present understanding of the oncogenic function of DEPDC1B in LIHC progression remains unclarified. Increasing studies provide possible mechanistic explanations for the relationship between high DEPDC1B expression and poor prognosis in other types of tumors. Some studies indicated that the downregulation of DEPDC1B expression could suppress cell proliferation by promoting apoptosis in malignant melanoma (Xu et al., 2019), bladder cancer (Lai et al., 2020), and glioblastoma (Chen et al., 2020). In non-small cell lung cancer, DEPDC1B could enhance cell migration and invasion through the activation of Wnt/β-catenin signaling, and this biological effect could be inhibited by the depletion of LEF1 or TCF4 (Yang et al., 2014). In oral carcinoma, however, due to disruption of HPV E2, which is a viral tumor suppressor and is known to downregulate the expression of DEPDC1B, highly expressed DEPDC1B could interact with RAC1 and result in cell invasion/metastasis (Ahuja and Singh, 2016). RAC1 which is a major component of Rho GTPase signaling, and is known to regulate actin cytoskeleton (participate in the early stage of autophagosome formation), could regulate the cell cycle, cellular growth, and mediate cell proliferation *via* NF-κB activation (Ehrlich et al., 2002; Bauer et al., 2007; Bosco et al., 2010; Saci et al., 2011). Furthermore, LC3 which is a famous autophagy marker could interact with SOS1 to inhibit the GEF activity and block the activation of RAC1, which could also be inhibited by exogenous expression of DEPDC1B. Thus, the RAC1 signaling pathway is mutually regulated by autophagy and DEPDC1B. A similar study further proved that DEPDC1B may affect the prognosis of patients with prostate cancer through the regulation of autophagy (Bai et al., 2017). DEPDC1B could regulate RAC1 activity by increasing GTP loading in RAC1 instead of affecting Rho A activities in normal or cancer cells (Su et al., 2014). More recently, Liu et al. reported that long noncoding RNA lncNB1 could interact with ribosomal protein RPL35 to enhance the synthesis of E2F1 protein, leading to DEPDC1B gene transcription. The GTPase-activating protein DEPDC1B then induces ERK protein phosphorylation and the stabilization of the N-Myc protein in neuroblastoma cells. Conversely, the downregulation of lncNB1 mitigates the clonogenic capacity of neuroblastoma cells *in vitro* and leads to tumor regression *in vivo* (Liu et al., 2019). The study strongly suggests an additional pathway of DEPDC1B to induce carcinoma.

## Conclusion

In summary, based on the Oncomine and TCGA databases, we have observed that high DEPDC1B expression is associated with poor prognosis in LIHC, suggesting that DEPDC1B could be a valuable diagnostic and prognostic marker in LIHC.

## Data Availability

The original contributions presented in the study are included in the article/Supplementary Material, further inquiries can be directed to the corresponding author.
