# Supplementary figures and images for: Identification and Validation of DEPDC1B as an Independent Early Diagnostic and Prognostic Biomarker in Liver Hepatocellular Carcinoma
Source: Front Genet. 2022 Jan 13;12:681809. doi: 10.3389/fgene.2021.681809 (PMC8793833; doi:10.3389/fgene.2021.681809)

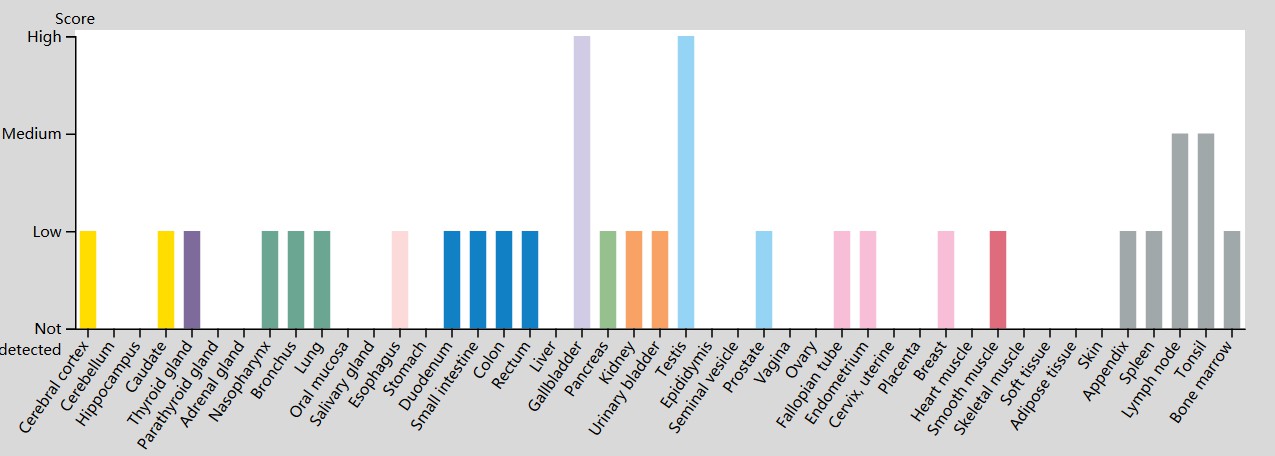

Supplement: Supplementary file 1 [file Image3.JPEG]

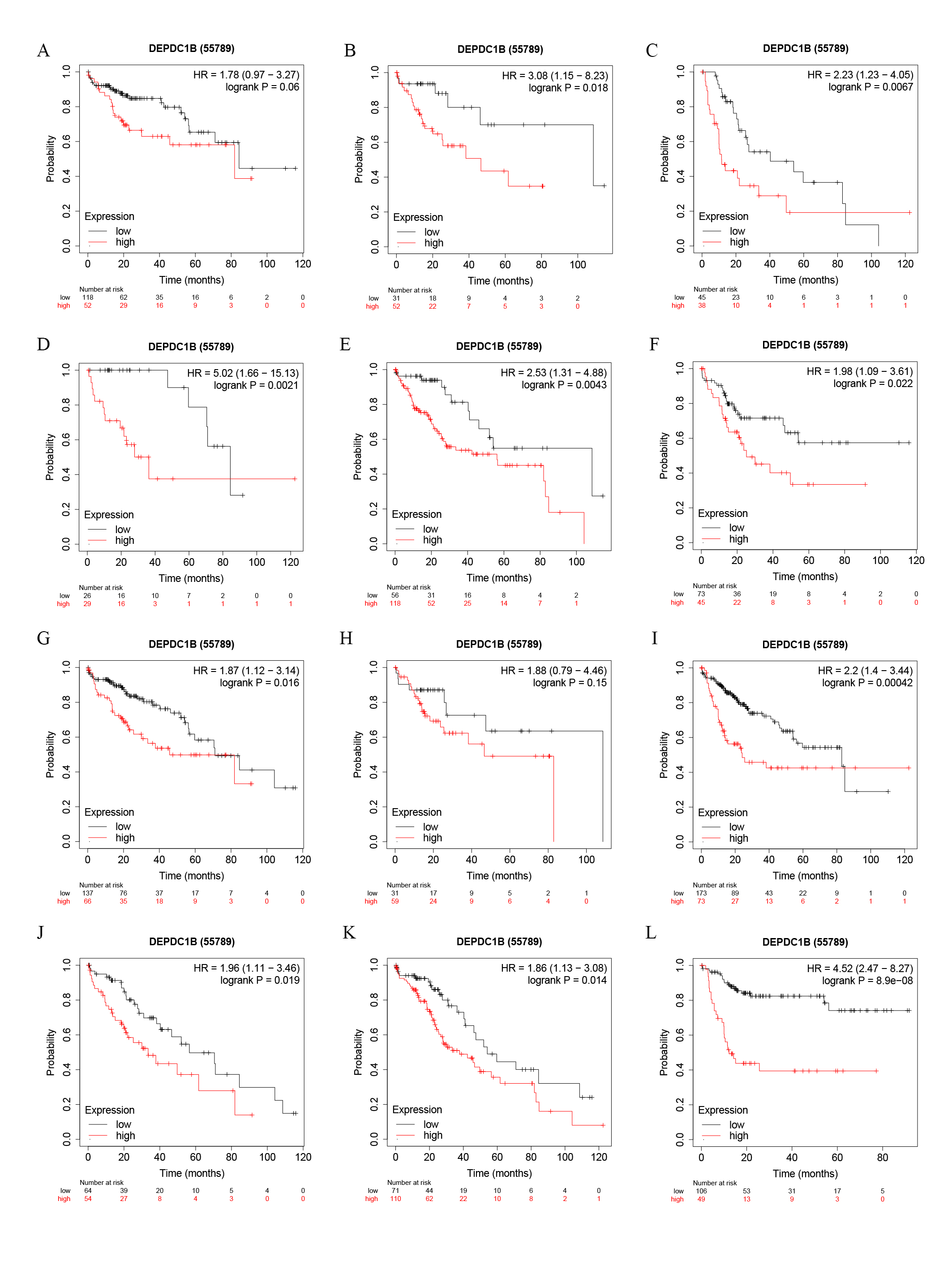

Supplement: Supplementary file 2 [file Image1.JPEG]

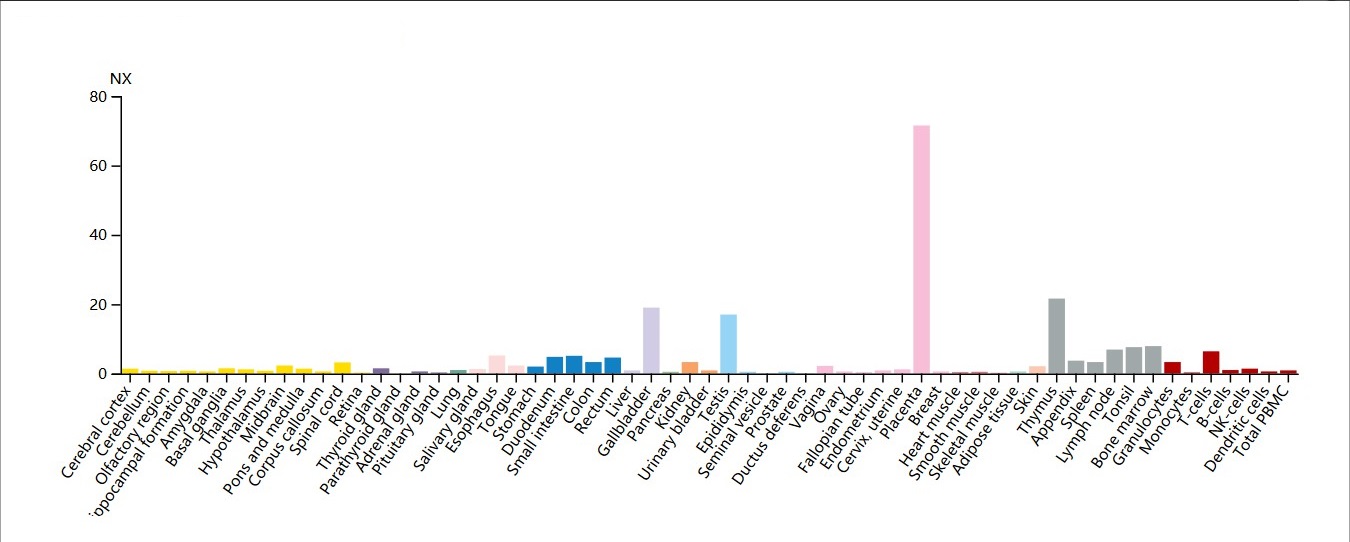

Supplement: Supplementary file 3 [file Image2.JPEG]
